# Evolving topological colour landscape unravels the final stages of pistachio nut development and the incidence of blank nuts

**DOI:** 10.1098/rsif.2025.0119

**Published:** 2025-09-24

**Authors:** Abdul-Hakeem Omotayo, Fushing Hsieh, Yiduo Wei, Paula Guzmán-Delgado, Giulia Marino, Barbara Blanco-Ulate

**Affiliations:** ^1^ Department of Statistics, University of California Davis, Davis, CA, USA; ^2^ Department of Plant Sciences, University of California Davis, Davis, CA, USA

**Keywords:** image colour processing, non-destructive assessment of nut maturity, categorical exploratory data analysis

## Abstract

Pistachio is a major nut crop worldwide; however, there is a lack of standardized non-destructive methods to effectively evaluate maturity and kernel filling for improved management and harvest timing. This study presents an image-based approach to determine pistachio nut maturation and blank kernel incidence by analysing the surface colour patterns of individual nuts at three time points during late development. We identified eight major hull colours to represent the full colour spectrum and applied principal component analysis to divide each nut into seven spatial sections. Within each section, we constructed eight colour-based feature variables (covariates) and associated them with a binary response variable indicating kernel presence or absence. We explored the specific response–covariate relationships at each developmental time point using a data-driven method called categorical exploratory data analysis, which identified key first-order and second-order feature-categories that link hull colour patterns with kernel status. These relationships were visualized using block-structured heatmaps, revealing consistent distinctions between filled and blank nuts. Based on these findings, we developed an algorithm with two main functions: (i) identifying a nut’s growth stage from its image for optimal harvest timing and (ii) estimating blank nut incidence for quality assessment and economic decision-making.

## 1. Introduction

**Figure 1.**
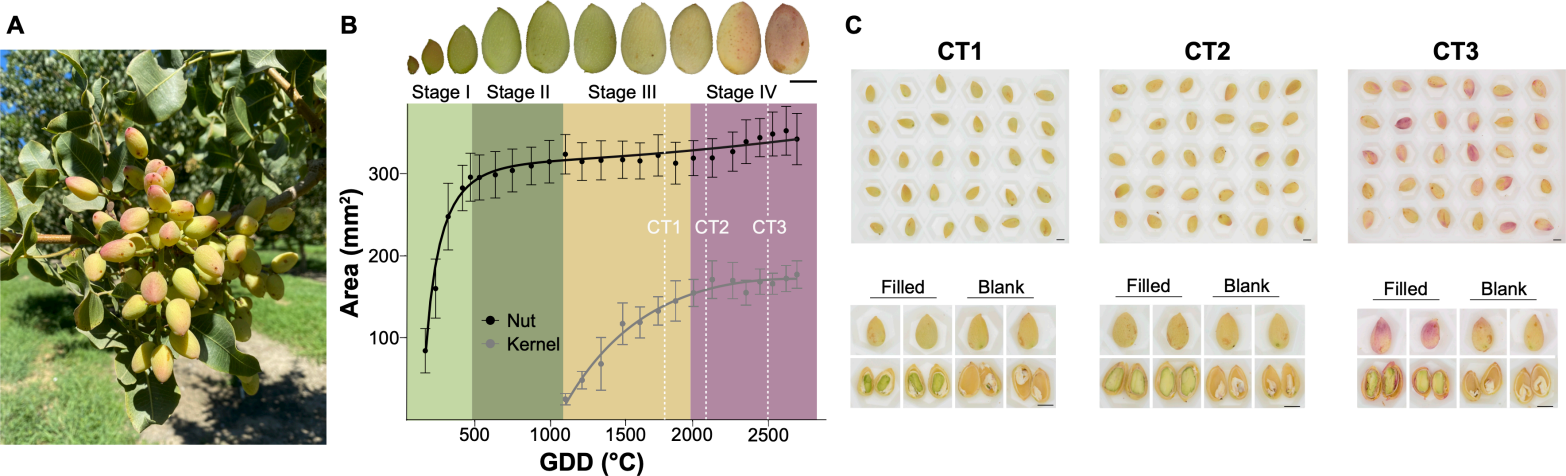
(A) A pistachio branch on a tree at critical time point two. (B) Pistachio development with images corresponding to the four stages categorized by physiological traits. The whole nuts and kernel sizes were measured from April through September 2019 in California as growing degree days (GDD) in °C, an accumulated heat unit. Three critical time points are in white dashed lines. (C) One plate of 30 pistachio nuts is shown for each critical time point, with images of two whole and opened nuts showing the kernel filled–blank status.

Pistachio (*Pistacia vera* L.) is a valuable and sustainable crop known for its resilience to climate change and high nutritional value (figure 1A). Although pistachios are often referred to as nuts, they are technically a type of fruit called a ‘drupe’, similar to peaches or mangoes. A pistachio has three main parts: the outer layer, named the hull (which includes the exocarp and mesocarp), the hard shell (the endocarp) and the kernel (the seed), which is the edible part. These components develop at different rates throughout the growing season and are influenced by factors such as temperature, crop load and the management of the crop by the growers.

After several years of studying the development of pistachio nuts, we discovered that changes in hull colour and texture are closely related to hull maturation and ripening [1]. Initially, the hulls are hard, green-yellow and firmly attached to the shells. As the nuts ripen, the hulls soften and change colour to pale yellow or pink, becoming more easily detached from the shells. These changes primarily occur during Stage IV of pistachio nut development, as illustrated in figure 1B. Additionally, we observed that specific patterns of hull colour, particularly red colouration at the tip of the nut, were more frequently seen in nuts without a kernel, a defect known as blank nuts.

In this study, we focus on three critical time points: critical time point one (CT1), indicating the completion of kernel filling at approximately 1800 growing degree days (GDD); critical time point two (CT2), when hull softening begins and the shell starts to split, around 2100 GDD; and critical time point three (CT3), corresponding to the commercial harvest time at about 2500 GDD (figure 1C). We collected images of the hulls of individual pistachio nuts in these critical developmental stages, with each image labelled according to the status of the kernel: filled or blank. Using this dataset, we developed a novel image-based analysis protocol involving two major computational steps. First, we transformed the hull images from each time point into a structured dataset that preserved relevant visual and biological information, including the hull colour patterns and the corresponding kernel status. Second, we applied the model-free categorical exploratory data analysis (CEDA) method to extract and interpret associations within each dataset. This enabled us to characterize how the hull colour features relate to the maturity of the nut and the development of the kernel. The resulting framework supports practical applications for determining optimal harvest timing, assessing blank kernel incidence for quality assessment, and evaluating the economic viability of the crop for the year.

## 2. Material and methods

### 2.1. Plant material and field setup

Pistachio samples were collected to develop a comprehensive image dataset of individual whole and halved nuts from ‘Golden Hills’, a widely planted California cultivar known for its early bloom, rapid nut maturation and high yield. Sampling was conducted in a commercial orchard located on the west side of Fresno County in California’s San Joaquin Valley. The orchard consisted of fully mature trees grafted onto UCB-1 rootstock and was managed according to standard California Pistachio Management guidelines [2].

Irrigation matched crop evapotranspiration, based on reference evapotranspiration data from the nearest public weather station and crop coefficients developed by Goldhamer [3], and was delivered via a micro-irrigation system. Mechanical pruning (topping and edging) was performed annually, and standard weed and pest control practices were followed. A winter cover crop was grown and mowed in spring, with the residue left as surface mulch. Fertilizers were primarily applied through fertigation. The soil type was classified as Panoche loam, based on USDA-NRCS soil maps.

Air temperature was monitored using a shielded *in situ* temperature and humidity sensor (Metos) positioned above the canopy. Daily average temperature data were used to calculate GDD with a base temperature of 7°C. GDD accumulation began on the date of full bloom, identified via time-lapse cameras installed in the orchard. Let GDD[n] be the GDD up to the nth day:


$$\text{GDD}[n]=\sum_{i=1}^{n}\max\left\{\frac{T_{\text{max},i}+T_{\text{min},i}}{2}-T_{\text{base}},\;0\right\},$$

where $T_{\text{max},i}$ and $T_{\text{min},i}$ are the maximum and minimum temperatures on day i, and $T_{\text{base}}=7^{\circ }\text{C}$ is the pistachio-specific baseline temperature. Eight representative trees were randomly selected, and inflorescences at similar developmental stages were tagged on eight branches per tree shortly after bloom. To minimize bloom-time effects on nut development, inflorescences were chosen uniformly from all four cardinal directions of each canopy, accounting for microclimatic variation such as light exposure and temperature. The three key developmental stages for imaging were defined by specific GDD accumulation thresholds.

### 2.2. Image acquisition and processing

#### 2.2.1. Background removing

**Figure 2.**
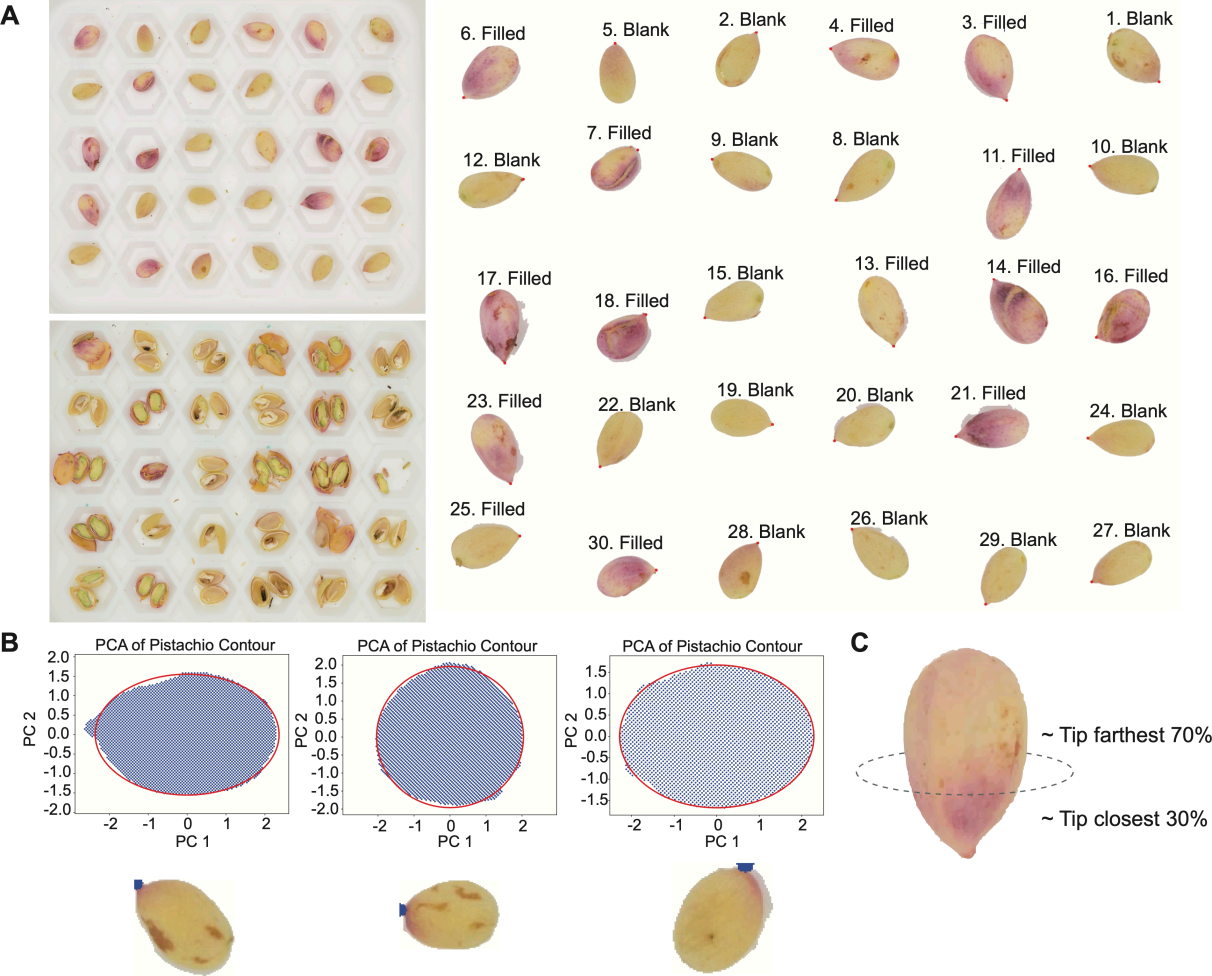
The workflow demonstrates the image processing, from image data to structured data. (A) The data extraction starts from the top photo and involves two steps, labelling and segmenting. The nuts were labelled from 1 to 30 from top to bottom, and the individual nut images were segmented. The bottom left photo is the same plate of nuts, with the corresponding positions as they are in the first photo; cut open to see if the kernel exists in each nut. Some of the image backgrounds were still retained after segmenting, but they were later excluded in the following steps of the colour analysis. The kernel status is then annotated onto the individual nut images. (B) Tip identification using principal component analysis (PCA). (C) Feature extraction based on the proximity of pixels to the tip.

The process for isolating and extracting individual pistachio nut images from a plate is illustrated in figure 2A. In the first step, image processing techniques are used to algorithmically detect and label all nuts present on the plate. To ensure consistent colour representation, we begin by applying chromatic adaptation to adjust the plate’s white background so that it approximates a natural white tone, following the algorithm outlined in [4]. Adaptive thresholding is then applied to effectively distinguish pistachios from the background.

In the second step, the thresholds obtained from the initial segmentation are used to generate contour approximations that remove redundant boundary points, enabling a tighter and more accurate fit around each nut. The outermost contours, which may include plate edges or background noise, are discarded, leaving only the contours corresponding to individual pistachio nuts. Based on these refined contours, each nut is isolated and saved as a separate labelled image for downstream analysis.

#### 2.2.2. Kernel status annotation

Each critical time point was represented by a set of pistachio nut images, with each set consisting of three plates of 90 nuts in total. Each plate includes one image of whole nuts for hull colour assessment and one image of open nuts. This shows the nuts’ corresponding positions for evaluating kernel status, which was categorized into two states: filled and blank. Thus, the raw data for this study consisted of two versions of nut images. These pictures were used for extracting individual nuts and annotating their kernel status, as shown in figure 2A.

### 2.3. Tip identification

Each isolated nut image was subsequently processed to identify the tip of the nut, which provided spatial coordinate references for mapping colour patterns. The tip detection method was adaptive to the shape characteristics of individual pistachio nuts. Each image was treated as an ensemble of pixels representing the nut surface. Representative examples of three processed nuts are shown in figure 2C. Each pixel bears five-dimensional measurements: (*x*,*y*) spatial coordinates for its location and (r,g,b) colour components in the RGB space. We observe that a pistachio nut is nearly elliptical, with a slightly pointed tip that characteristically protrudes. This recognition leads us to apply principle component analysis (PCA) on its pixel-coordinate ensemble and calculate the lengths of the long-axis and short-axis of the approximating ellipse. We took the centre of this ellipse as the centre of the pistachio nut’s image. Then, we rotate the image’s (*x, y*) spatial coordinates such that the long-axis is horizontal, as shown in figure 2C. With respect to the horizontal axis, there are two extreme coordinates on the two sides of the designated centre. The extreme point, having a longer distance to the centre, is identified as the tip of the pistachio. The identified tip on the transformed PCA is matched to the corresponding point on the pistachio, thus identifying the actual tip. This simple tip-identification approach enjoys nearly perfect reliability.

The significance of identifying the tip of the nut is both scientific and practical. As mentioned earlier, our observations indicate that the colour surrounding a pistachio tip is a key factor in determining whether it contains a kernel or is empty. Growers have also empirically confirmed these observations. To validate our hypothesis, we needed to extract quantitative features based on the colour content and analyse the directional associative patterns of these features.

### 2.4. Spectrum of major colours

**Figure 3.**
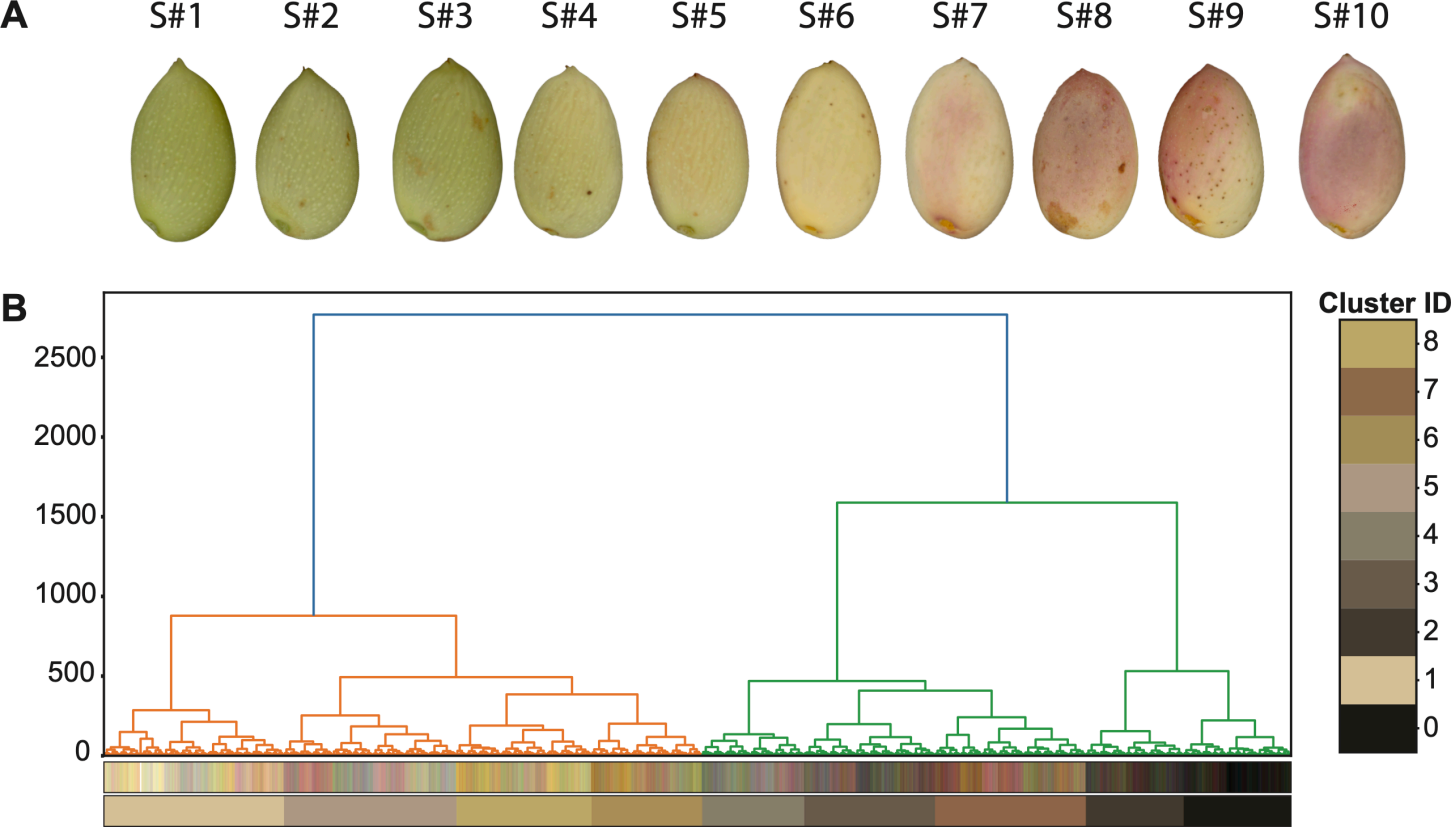
(A) A selected series of pistachio nuts for building a reference colour-spectrum and individual pistachio image captured by OpenCV Python Package [5]. (B) HC-tree based on the collection of RGB coordinates of all pixels in the 10 individual pistachio images. The nine major colours are marked according to the nine branches at the bottom of the tree.

We then created a comparative analysis base for the major colours in pistachio hulls. Using the hierarchical clustering (HC) tree of RGB coordinates collected from the 10 individual pistachio nuts, as illustrated in figure 3B, we selected nine colour clusters or profiles. We excluded the black colour cluster because it represents the background and outer edge of the nut, which was included solely to enhance the extraction of individual pistachio nuts. The remaining eight colour clusters were designated as the major colours, serving as the standard spectrum of colour content for defining features associated with all individual pistachio nuts analysed at three critical time points.

It is important to note that these eight major colours were defined by the members of eight branches, rather than by their branch-specific averaged RGB values displayed at the bottom of the HC-tree in figure 3B. Essentially, a major colour represents a branch-specific collection of RGB coordinates from the pixels.

Comparing the clusters captured in figure 3B and the range of colour of pistachios in figure 3A shows how visually representative these eight colour profiles are.

### 2.5. Feature space construction

We constructed a collection of covariate features derived from three different areas of each pistachio nut: (i) the entire hull, (ii) part of the hull near the nut tip, and (iii) part of the hull near the bottom. Specifically, a set of eight features would be defined for each of the seven areas of a pistachio nut according to the following feature-defining protocol. The seven areas include: the ‘full’, tip-closest-k and tip-farthest-k with k=30,50 and 70. Here, k indicates the percentage of pixels. These features are generated as illustrated in figure 2B.

#### 2.5.1. Feature defining protocol

— Upon a targeted area, all RGB coordinates of all pixels were classified using Euclidean distance to the nine colour profiles. This excluded the black colour that is seen around the edge of the individual nut image.
— The proportions of pixels were counted from this targeted area with respect to eight colour profiles to form an 8-dim vector.
— The eight vector components were defined as a set of eight features specific to this targeted area.

**Figure 4.**
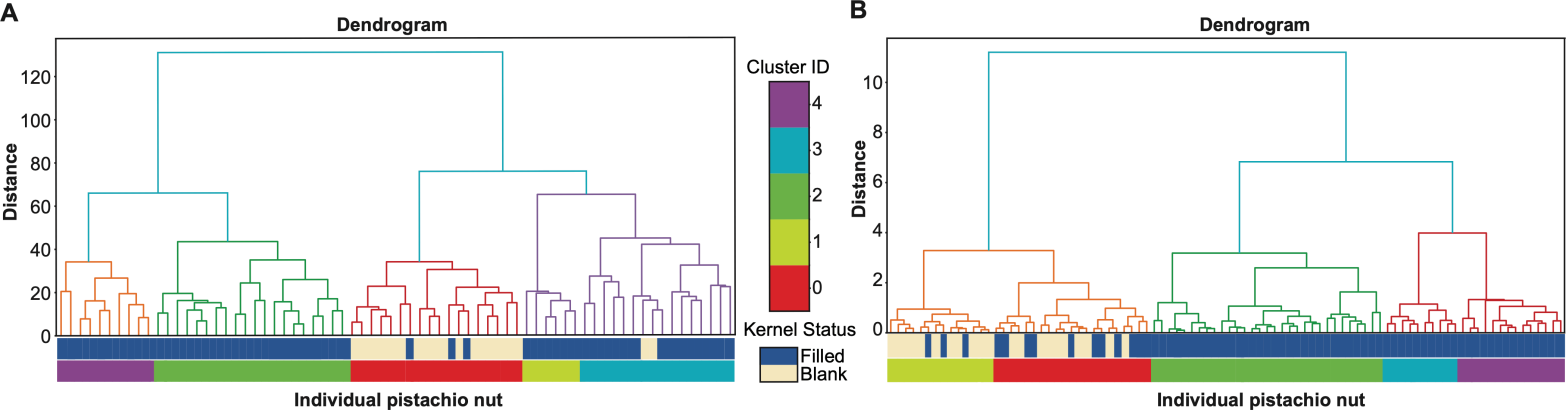
Two HC-trees of CT3 based on all pistachio nuts' full covariate vectors of all one-features (A) and all two-features (B).

This feature-defining protocol enabled the definition of 56 features per nut, derived from the major colour proportions across three spatial regions of each pistachio. As a result, each nut was represented by a 56-dimensional feature vector. An example HC tree constructed from 90 such feature vectors is shown in figure 4. Clustering was performed using Euclidean distance and the Ward-D2 linkage method. Ward-D2 was selected because it minimizes within-cluster variance, resulting in clusters of moderate and relatively balanced size. This contrasts with the tendencies of single linkage, which often produces a ‘chaining’ phenomenon with a few large clusters, and complete linkage, which tends to over-segment the data into many small, tight clusters. Previous studies have shown that Ward-D2 generally performs as well as or better than complete linkage in terms of cluster quality [6]. It is noted that the three clusters, colour-coded as Green, Purple and Blue, are exclusively filled nuts, while the other two clusters, colour-coded as Yellow and Red, contain a mix of filled and blank nuts.

Based on these 56 1D-features, we can generate (562)=1540 feature-variables of order-2. That is, an individual pistachio nut can be further represented by 1540-dim 2-feature-categories. Again, applying the HC algorithm resulted in an HC-tree with a visible degree of improvement in containing blank nuts by separating them from the filled nuts, as shown in figure 4B. It is evident that at the two branches’ HC-tree level, the right branch is exclusively filled nuts, while the left branch contains all blank nuts and some filled ones as a minority. We further improved this result in the next section by employing the CEDA paradigm.

We examined current popular methodologies for automatic feature selection applied to image data. By ignoring previous knowledge and experience in pistachio nut development, we focused on three approaches: (A) Gabor filters [7] are linear filters employed for texture analysis, edge detection and feature extraction; (B) grey level co-occurrence matrix (GLCM) [8], a statistical method used to examine the texture of an image by analysing the spatial relationships between pixels. It calculates how often pairs of pixels with specific values and spatial relationships occur, providing insights into the image’s texture; (C) histogram of oriented gradients (HOG) [9] is a feature descriptor used in computer vision and image processing for to extract features from image data. It works by counting occurrences of gradient orientations in localized portions of an image, capturing the shape and structure of objects.

**Figure 5.**
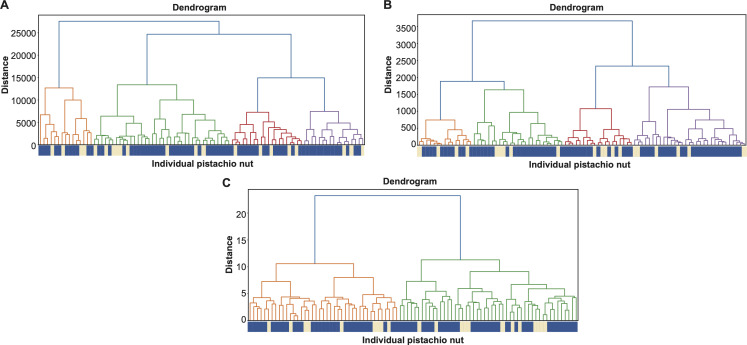
HC-trees of three automatic feature selections: (A) Gabor; (B) GLCM; (C) HOG.

We applied these three approaches to the dataset of CT3, and their 1-feature results via HC-tree format are reported in figure 5. Although these results are far worse than those reported in figure 4A.

Automatically created features from a collection of images might sound very convenient. However, as far as selecting informative features is concerned, they are not suited for scientific purposes in general. In any scientific study, the goal is too specific, and the knowledge domain and experiences are critical to achieving a performant solution for any automatic feature-creating algorithm that was designed for general purposes.

### 2.6. Categorical exploratory data analysis paradigm

Before applying our computational paradigm (CEDA), we filtered the initial set of 56 features at each of the three critical time points to remove non‑informative features. Specifically, we eliminated any features that exhibited no variation (i.e. were constant) or nearly no variation across samples. In practice, we discarded features whose sample standard deviation was less than 0.01. Here, each feature $f_{i,t}$ is defined as the proportion of hull pixels in a major colour category f at time t (so $0\leq f_{i,t}\leq 1$), and the standard deviation was computed across these normalized proportions for all N samples at that time point. This threshold effectively removes features that are all zero or all the same, which often indicate the absence of a particular major colour at that time point. The remaining features—those with sufficient variability—were then used in subsequent analyses. The remaining viable features are recorded in table 1. For instance, major colour 8 in the tip-Closest-30 region remained informative (i.e. standard deviation is greater than or equal to 0.01) across all three time points, suggesting a stable contrast pattern in that spatial band. By contrast, major colour 5 in the tip-Closest-30 region was only informative at CT3, indicating a late‐emerging colour change. These retained features formed the input to our subsequent CEDA, ensuring that we focused on region-colour signals with genuine temporal dynamics rather than noise.

**Table 1.** Major colour‐category features that passed our variability threshold at each critical time point (i.e. $\mathrm{SD}(f_{.,t})\geq 0.01$). Each cell lists the time points (CT1, CT2, CT3) at which that major colour for the hull's region remained informative and was therefore retained for CEDA.

features/colour-codes	1	2	3	4	5	6	7	8
full	CT2,3				CT2,3	CT1,2		CT1,2,3
tip-Closest-30	CT2,3				CT3	CT1,2,3	CT3	CT1,2,3
tip-Closes-50	CT2,3				CT3	CT1,2,3	CT3	CT1,2,3
tip-Closest-70	CT2,3				CT3	CT1,2	CT3	CT1,2,3
tip-Farthest-30	CT1,2,3				CT2,3	CT1,2		CT1,2,3
tip-Farthest-50	CT2,3				CT2,3	CT1,2		CT1,2,3
tip-Farthest-70	CT2,3				CT2,3	CT1,2	CT1	CT1,2,3

#### 2.6.1. Categorization of features based on major colour proportion in CT1

We first explained the protocol used for categorizing a feature related to the proportion of major colours. Take the feature $tip-closest-50_{8}$ as an example, which is the feature defined by the proportion of pixels having major colour code 8, located within 50% of the nut image, starting from the tip. This feature is significant based on our expertise as plant scientists, as well as insights from pistachio growers who understand the processes of nut maturity and ripening. We expected this feature to provide important associative information on the status of the nut kernel, whether it is blank or filled. The kernel status yields the binary response variable Y. A simple way of revealing such associative information is to categorize $tip-closest-50_{8}$ into a categorical variable, and then see the associative pattern from the contingency table of this categorized variable and Y. Here, we demonstrate the fact that effectively categorizing a continuous feature is critically essential for revealing its associative patterns. We gave two categorization approaches, naive and natural, and compared their effectiveness.

First, an intuitive categorizing approach is to divide the range of $tip-closest-50_{8}$ into two bins. These two bins are created by using the median as the cut-off. The binary codes: 0 and 1, indicate being smaller or larger than the median, respectively. The categorized feature, denoted as ${\hat{tip-closest-50}}_{8}$, gives rise to a 2×2 contingency table $HCT[{\hat{tip-closest-50}}_{8},Y]$ as shown in table 2. The directional associative pattern of ${\hat{tip-closest-50}}_{8}$ to Y is established in a row-by-row fashion of locality nature, not a weighted one in a marginal fashion of global nature. While the latter is frequently referenced in literature, its ambiguity poses a risk of misunderstanding, as some rows may have entirely contradictory associations.

**Table 2.** Contingency table $HCT[{\hat{tip-closest-50}}_{8},Y]$.

${tip-\hat{closest}-50}_{8}/Y$	blank	filled	row-sums	entropy
0	0	46	46	0.0
1	9	35	44	0.50664
col-sum	9	81	90	0.32508

The detection of the associative pattern is performed by comparing the row entropy (or odds) with the baseline entropy (or odds) of the column sum vector, which is an entropy (or odds) without having any information on the covariate ${\hat{tip-closest-50}}_{8}$. The baseline entropy and odds are 0.32508 and $\frac{1}{9}$. For the row of ${\hat{tip-closest-50}}_{8}=0$, its entropy is calculated as 0.0. For the row of ${\hat{tip-closest-50}}_{8}$, the entropy is 0.50664. Given that the two rows of counts differ from the column-sum vector, we intuitively expect that both rows indicate significant associative patterns. We can computationally confirm this intuition.

We adopted a Kolmogorov [10] randomness framework to simulate two ensembles of contingency tables for $HCT[{\hat{tip-closest-50}}_{8},Y]$. The *alternative ensemble* captures the observed column-wise variability by sampling each column from a multinomial distribution that matches its observed counts. The *null ensemble* enforces independence by sampling each column from the multinomial distribution defined by the marginal proportions of the covariate and the response. Comparing the entropy (or odds) distributions of corresponding rows between these ensembles, where their overlap quantifies the minimum sum of Type I and II errors, allows us to detect significant associative patterns.

**Figure 6.**
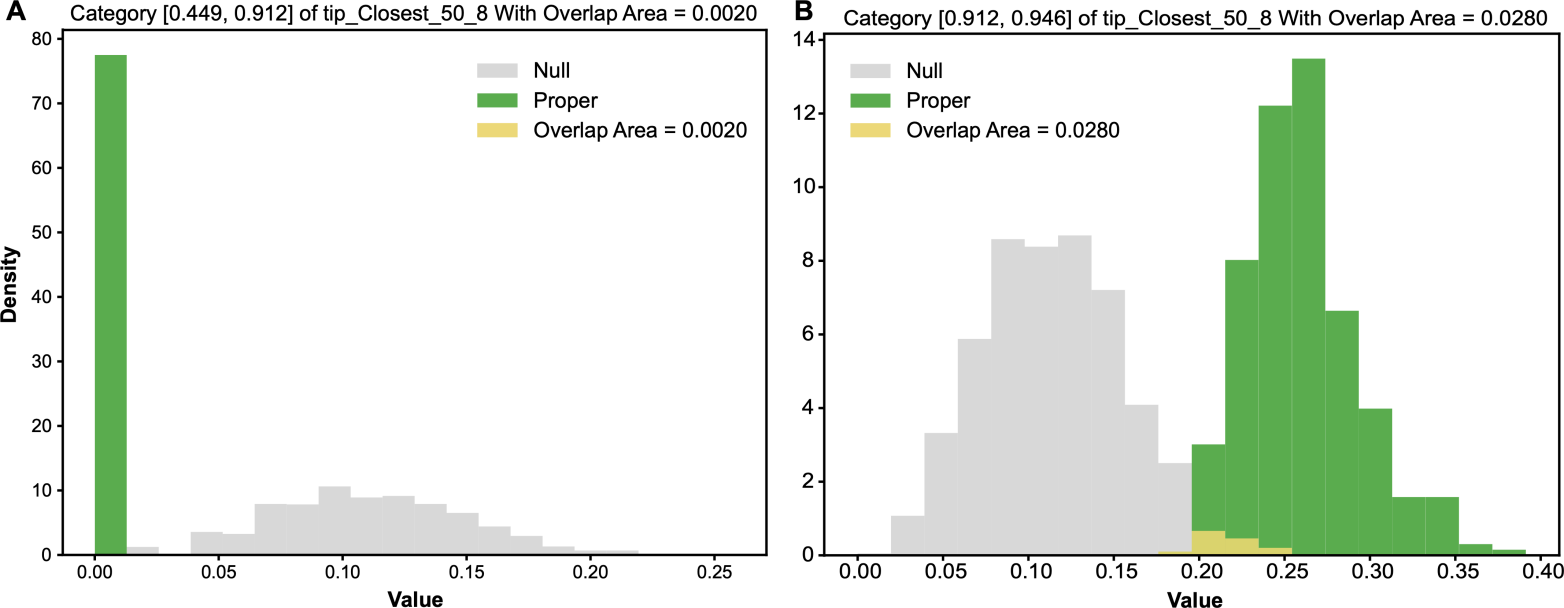
Two pairs of alternative-vs-null entropy distributions: (A) row of ${\hat{tip-closest-50}}_{8}=0$; (B) row of ${\hat{tip-closest-50}}_{8}=1$. Overlapping area as the minimum sum of Type-I and Type-II errors are marked.

**Figure 7.**
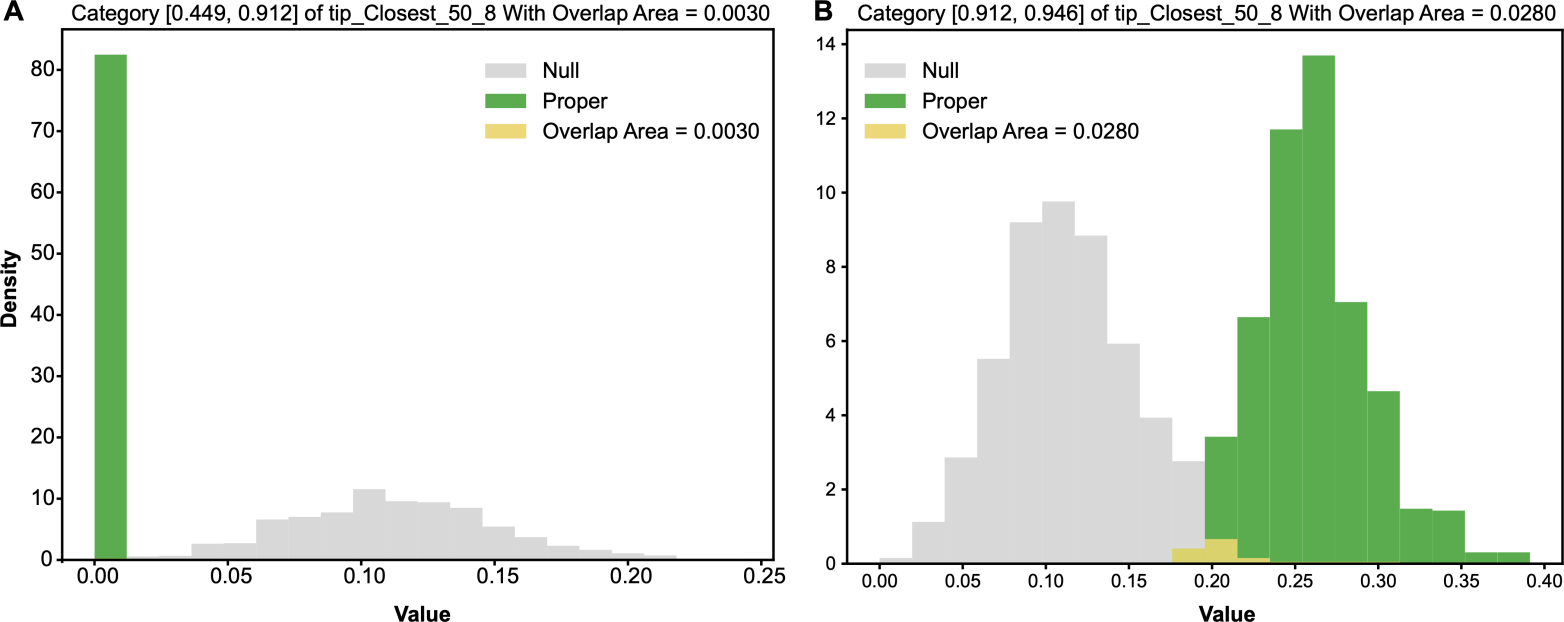
Two pairs of alternative-vs-null odds distributions: (A) row of ${\hat{tip-closest-50}}_{8}=0$; (B) row of ${\hat{tip-closest-50}}_{8}=1$. The overlapping area, which is the minimum sum of Type-I and Type-II errors, is marked.

Based on the alternative ensemble, the row of ${\hat{tip-closest-50}}_{8}=0$ has an entropy distribution. Likewise, the row of ${\hat{tip-closest-50}}_{8}=0$ has an entropy distribution from the null ensemble. We compared alternative-vs-null entropy distributions to detect whether the row of ${\hat{tip-closest-50}}_{8}=0$ gives rise to a significant associative pattern, as shown figure 6A. These two entropy distributions have an overlapping area, which is equal to the minimum sum of Type-I and Type-II errors, evaluated as being equal to 0.0. With respect to any chosen threshold, this 0.0 overlapping area strongly indicates that the 1-feature category ${\hat{tip-closest-50}}_{8}=0$ indeed gives rise to a very significant associative pattern. Likewise, the overlapping area for figure 6B is calculated as 0.0280. So, this 1-feature category ${\hat{tip-closest-50}}_{8}=1$ also gives rise to a very significant associative pattern with respect to most of the chosen thresholds. By contrast, the odds of blank ${\hat{tip-closest-50}}_{8}=0$ is calculated as $\frac{0}{46}$ compared with the marginal odds of blank $\frac{9}{81}$. Since the logarithm function is not involved in odds calculations, odds are evidently advantageous to give rise to bell-shape distributions based on the alternative and null ensembles of contingency tables; see figure 7.

**Figure 8.**
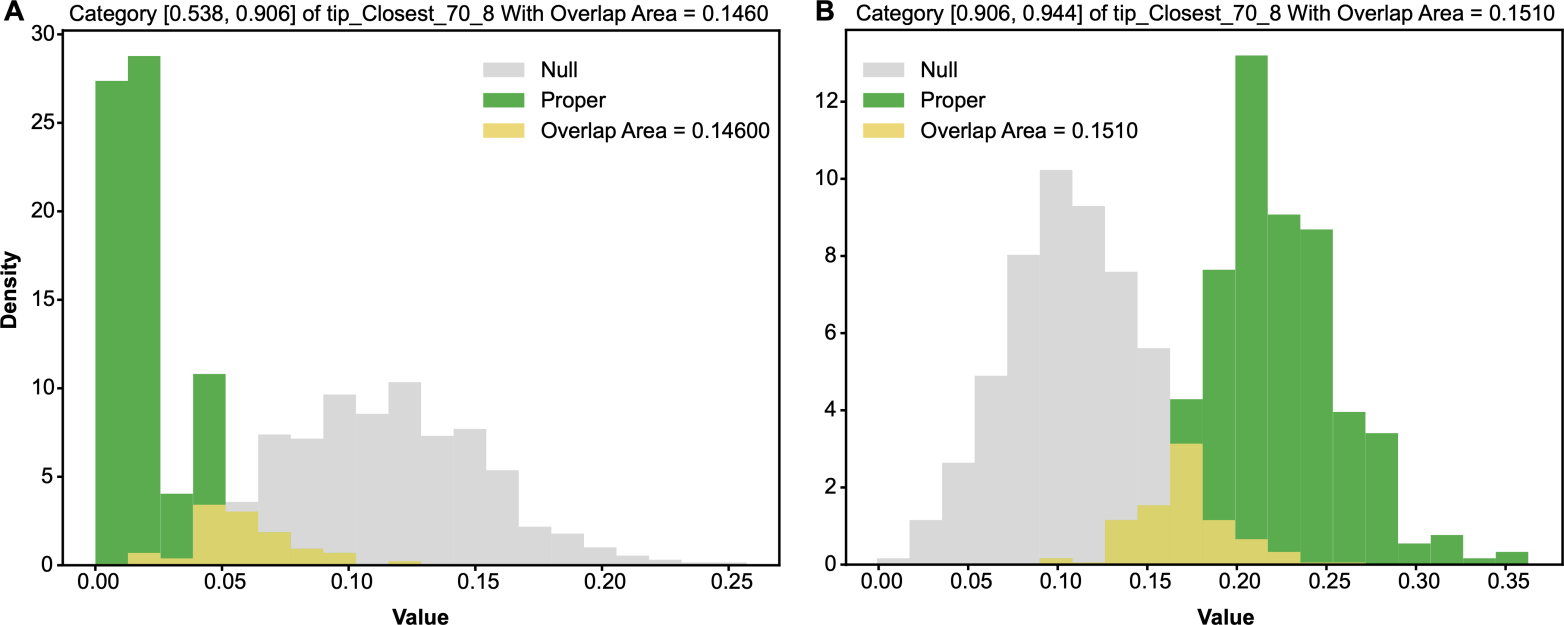
Two pairs of alternative-vs-null entropy distributions: (A) row of ${\hat{tip-closest-70}}_{8}=0$; (B) row of ${\hat{tip-closest-70}}_{8}=1$. The overlapping area, which is the minimum sum of Type-I and Type-II errors, is marked.

**Figure 9.**
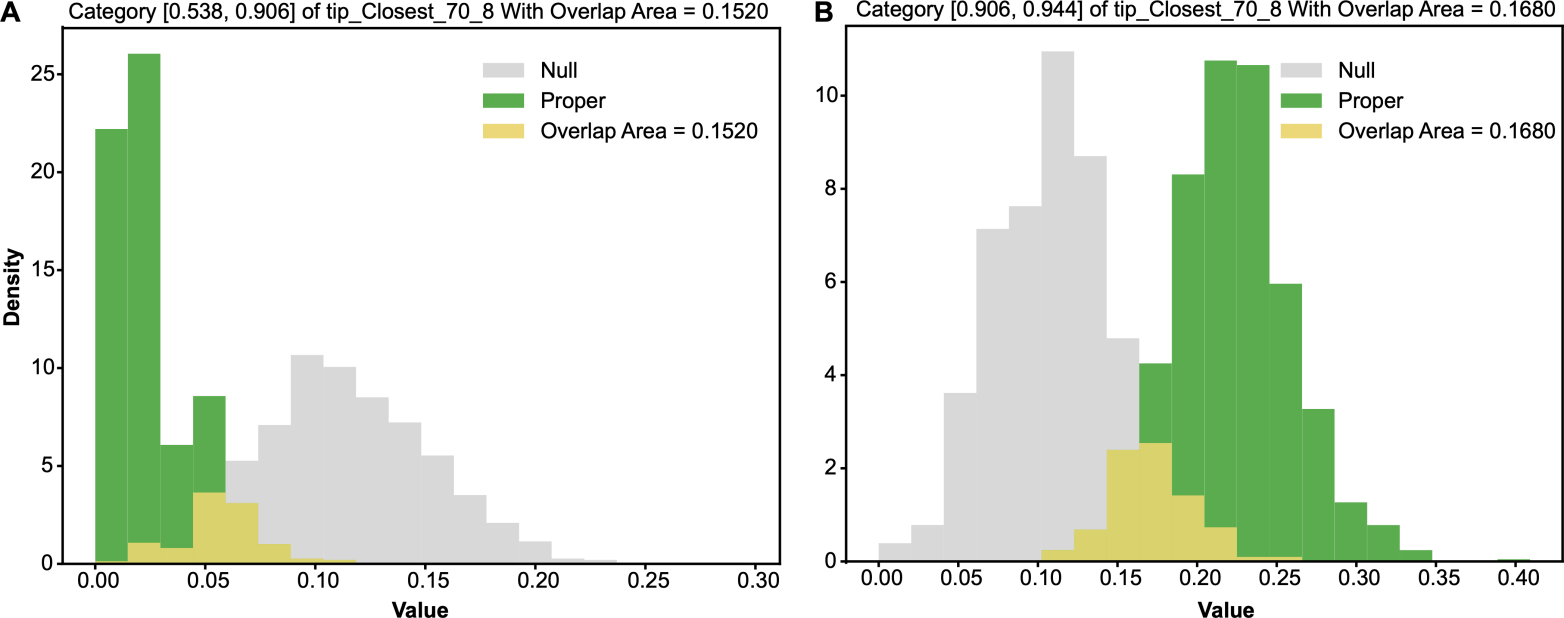
Two pairs of alternative-vs-null odds distributions: (A) row of ${\hat{tip-closest-70}}_{8}=0$; (B) row of ${\hat{tip-closest-70}}_{8}=1$. The overlapping area, which is the minimum sum of Type-I and Type-II errors, is marked.

In this CT1 period, we also found another important feature: $tip-closest-70_{8}$, which gives rise to two major 1-feature-categories if we set the threshold at 0.2. This feature is also of the same major colour-code 8. The contingency table of its categorized version ${\hat{tip-closest-70}}_{8}$ and Y: $HCT[{\hat{tip-closest-70}}_{8},Y]$, is given in table 3. The two major 1-feature categories: ${\hat{tip-closest-70}}_{8}=0$ and ${\hat{tip-closest-70}}_{8}=1$, are confirmed by their own pair of alternative-vs-null entropy distributions, as shown in the panels of figure 8 and by their odds distributions in figure 9.

**Table 3.** Contingency table $HCT[{\hat{tip-closest-70}}_{8},Y]$ .

${\hat{tip-closest-70}}_{8}-vs-Y$	blank	filled	row-sums	entropy
0	1	44	45	0.10656
1	8	37	45	0.46800
col-sum	9	81	90	0.32508

To make clear how we read any covariate–response link in our framework, we represent each association with a two-by-two contingency table as shown in table 3. In such a table, the column sums give the marginal distribution of the response (empty versus filled kernels), while each row is one feature category of the covariate and encodes the conditional distribution of the response for that category. Comparing a row’s conditional‐distribution vector with the marginal‐distribution vector then yields the associative relationship information for that feature category.

#### 2.6.2. Categorization of features based on major colour proportion in CT2

**Figure 10.**
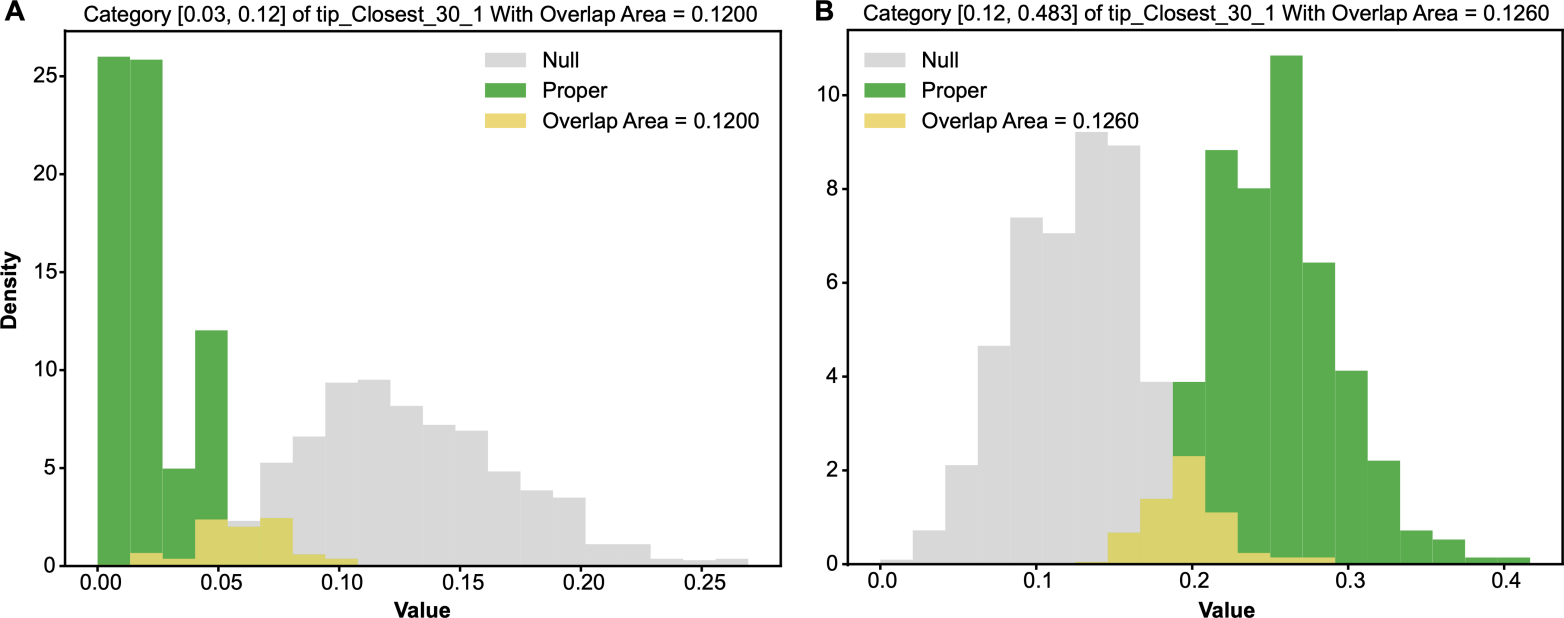
Two pairs of alternative-vs-null entropy distributions: (A) row of ${\hat{tip-closest-30}}_{1}=0$; (B) row of ${\hat{tip-closest-30}}_{1}=1$. The overlapping area, which is the minimum sum of Type-I and Type-II errors, is marked.

**Figure 11.**
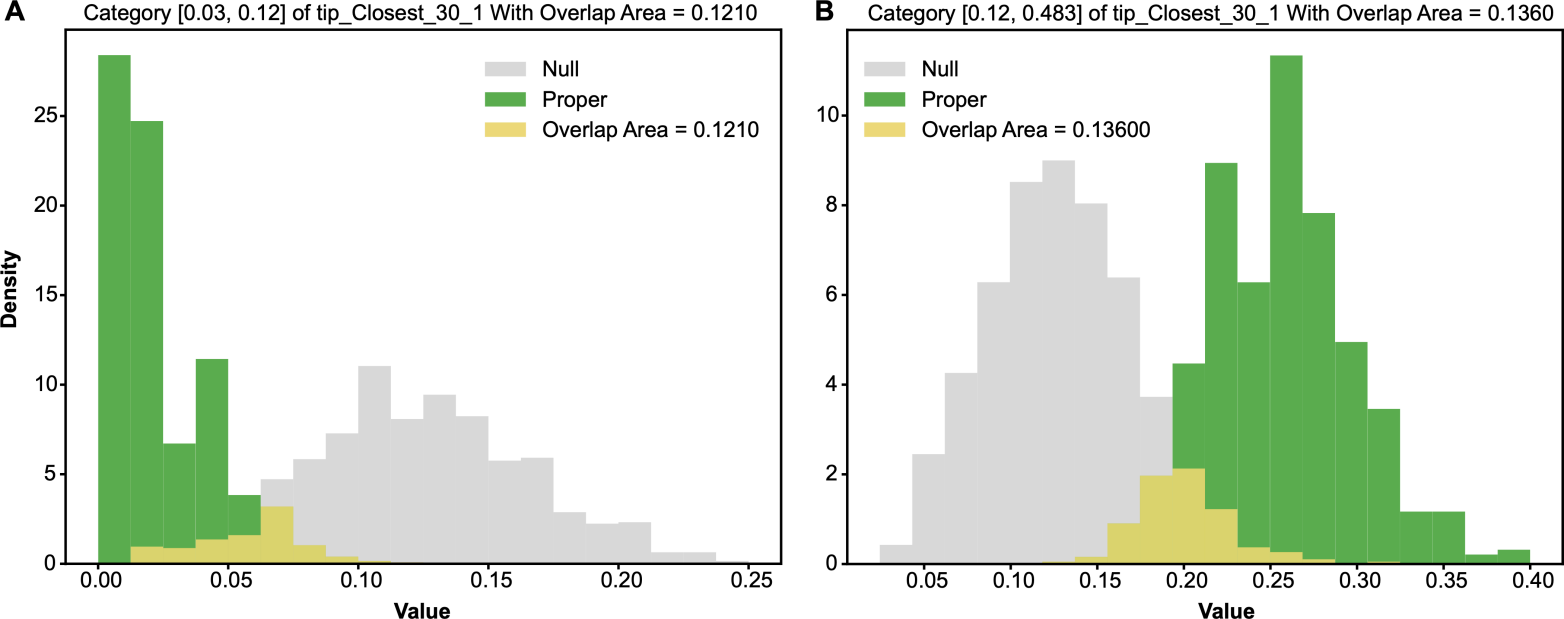
Two pairs of alternative-vs-null odds distributions: (A) row of ${\hat{tip-closest-30}}_{1}=0$; (B) row of ${\hat{tip-closest-30}}_{1}=1$. The overlapping area, which is the minimum sum of Type-I and Type-II errors, is marked.

In this CT2 period, we found only one important feature: $tip-closest-30_{1}$, which gave rise to two major 1-feature-categories if we set the threshold at 0.1. This feature is of the major colour code 1. The contingency table of its categorized version ${\hat{tip-closest-30}}_{1}$ and Y: $HCT[{\hat{tip-closest-30}}_{1},Y]$ is given in table 4. The two major 1-feature categories, ${\hat{tip-closest-30}}_{1}$= 0 and ${\hat{tip-closest-30}}_{1}$= 1, are confirmed by their pairs of alternative-vs-null entropy and odds distributions, as shown in figures 10 and 11, respectively.

**Table 4.** Contingency table $HCT[{\hat{tip-closest-30}}_{1},Y]$ in CT2.

${\hat{tip-closest-30}}_{1}-vs-Y$	blank	filled	row-sums	entropy
0	1	44	45	0.10656
1	9	36	45	0.50040
col-sum	10	80	90	0.34883

However, if we set the threshold to 0.3, then we would be able to find almost 20 major 1-feature-categories, as will be seen in §3.

#### 2.6.3. Categorization of features based on major colour proportion in CT3

We applied the same categorizing guideline discussed in CT1 for the feature $full_{1}$ in CT3. We used its median as the threshold. As seen in the contingency table $HCT[{\hat{full}}_{1},Y]$ in table 5, this ${\hat{full}}_{1}$ in CT3 is rather informative. Out of 21 blank pistachios, 19 fell into category-0, defined by the median with a range [0.045,0.411], while only two blank pistachios fell into category-1, defined by its range [0.411,0.899]. The two odds of blank in the two categories are calculated as $\frac{19}{26}=0.73077$ and $\frac{2}{43}=0.04651$ in comparison with the marginal odds of Y: $\frac{21}{69}=0.30435$, which is calculated from the column-sum vector without conditioning on categories of ${\hat{full}}_{1}$.

**Table 5.** Contingency table $HCT[{\hat{full}}_{1},Y]$ in CT3.

${\hat{full}}_{1}-vs-Y$	blank	filled	row-sums	entropy
0	19	26	45	0.68098
1	2	43	45	0.181812
col-sum	21	69	90	0.54327

**Figure 12.**
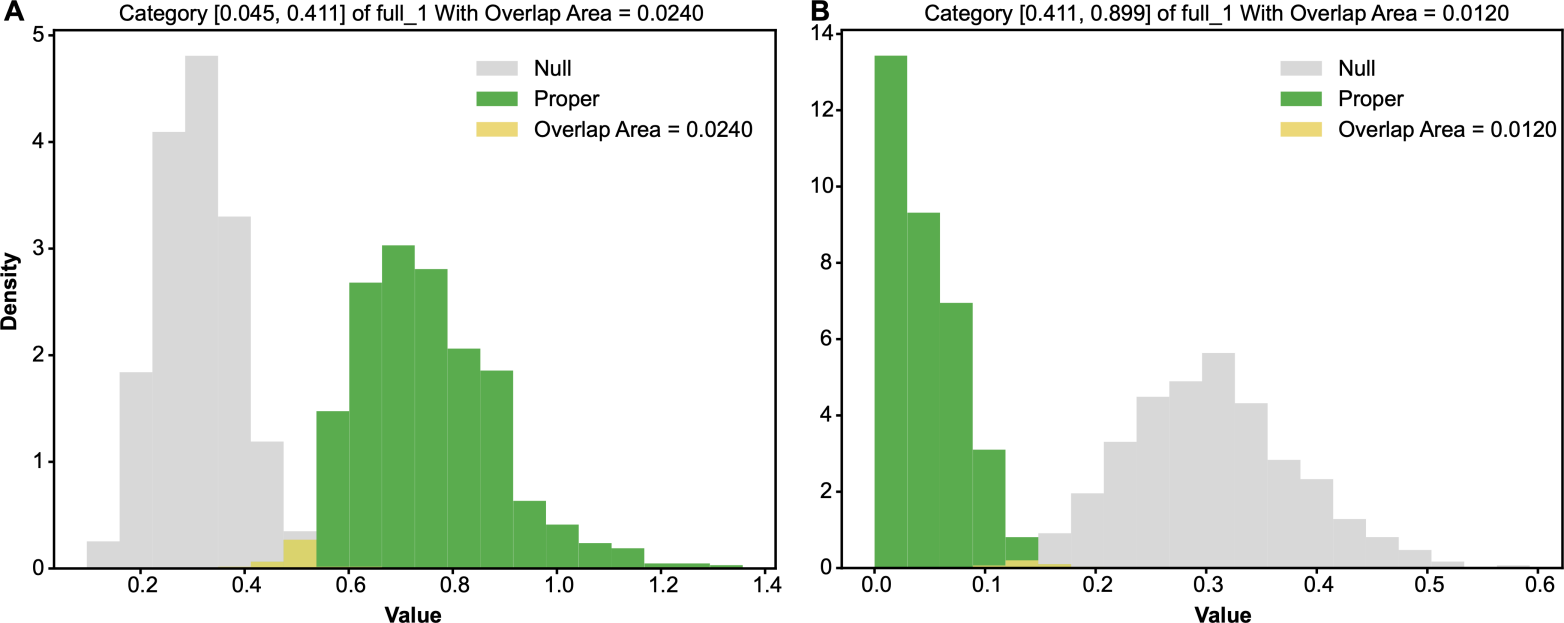
Two pairs of alternative-vs-null entropy distributions: (A) row of ${\hat{full}}_{1}=0$; (B) row of ${\hat{full}}_{1}=1$, at CT3.

**Figure 13.**
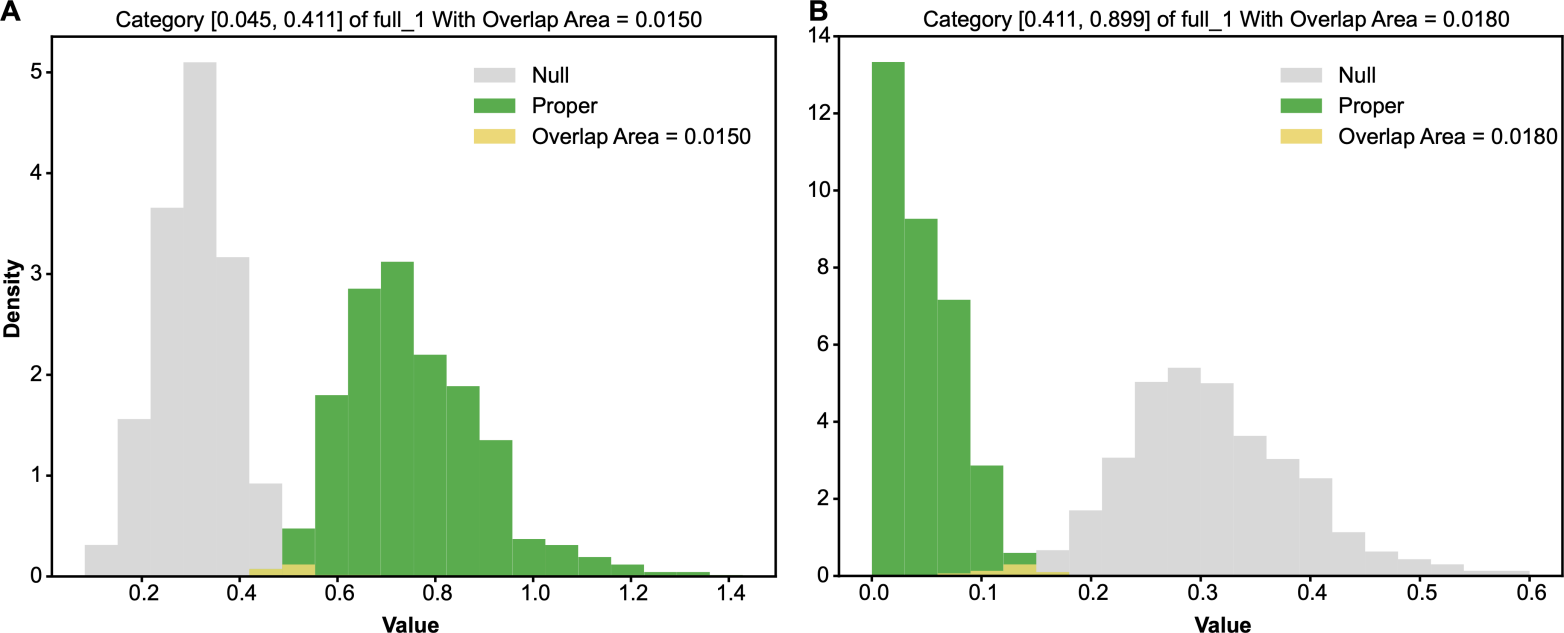
Two pairs of alternative-vs-null odds distributions: (A) row of ${\hat{full}}_{1}=0$; (B) row of ${\hat{full}}_{1}=1$, at CT3.

From the entropy perspective, it is not clear whether the category ${\hat{full}}_{1}=0$ could give rise to a significant entropy difference relative to that of marginal ${\hat{full}}_{1}$ or not. However, from a odds perspective, the odds of the category ${\hat{full}}_{1}=0$ calculated as $\frac{19}{26}=0.73077$ is significantly larger than the overall odds $\frac{21}{69}=0.30435$. On the other hand, we see the category ${\hat{full}}_{1}=1$ gives an evidently distinct entropy and odds. In fact, both categories lead to significant conditional entropy and odds differences from the marginal ones. As shown in figure 12, the two pairs of alternative-vs-null entropy distributions’ overlapping areas were calculated as: 0.0340 and 0.02. As such, with respect to the choice of threshold 0.1, both categories ${\hat{full}}_{1}=0$ and ${\hat{full}}_{1}=1$ are major 1-feature-categories. Likewise, as shown in figure 13, the two pairs of alternative-vs-null odds distributions’ overlapping areas are calculated as 0.0200 and 0.0140. The overlapping areas calculated based on the two pairs of alternative-vs-null odds distributions were slightly smaller than those based on the entropy distributions. This fact indicates that odds distributions are more stable than entropy ones in this situation. Therefore, we adopted the odds when calculating the overlapping areas between the alternative and null distributions.

It is worth reporting that we identified 8 major 1-feature-categories from 4 of the 8 features of major colour proportions: $full_{1}$, $full_{5}$, $full_{7}$ and $full_{8}$, derived from the full hull of pistachio nuts.

For further technical details on the computational steps and heatmap construction, refer to the electronic supplementary material.

## 3. Results

**Figure 14.**
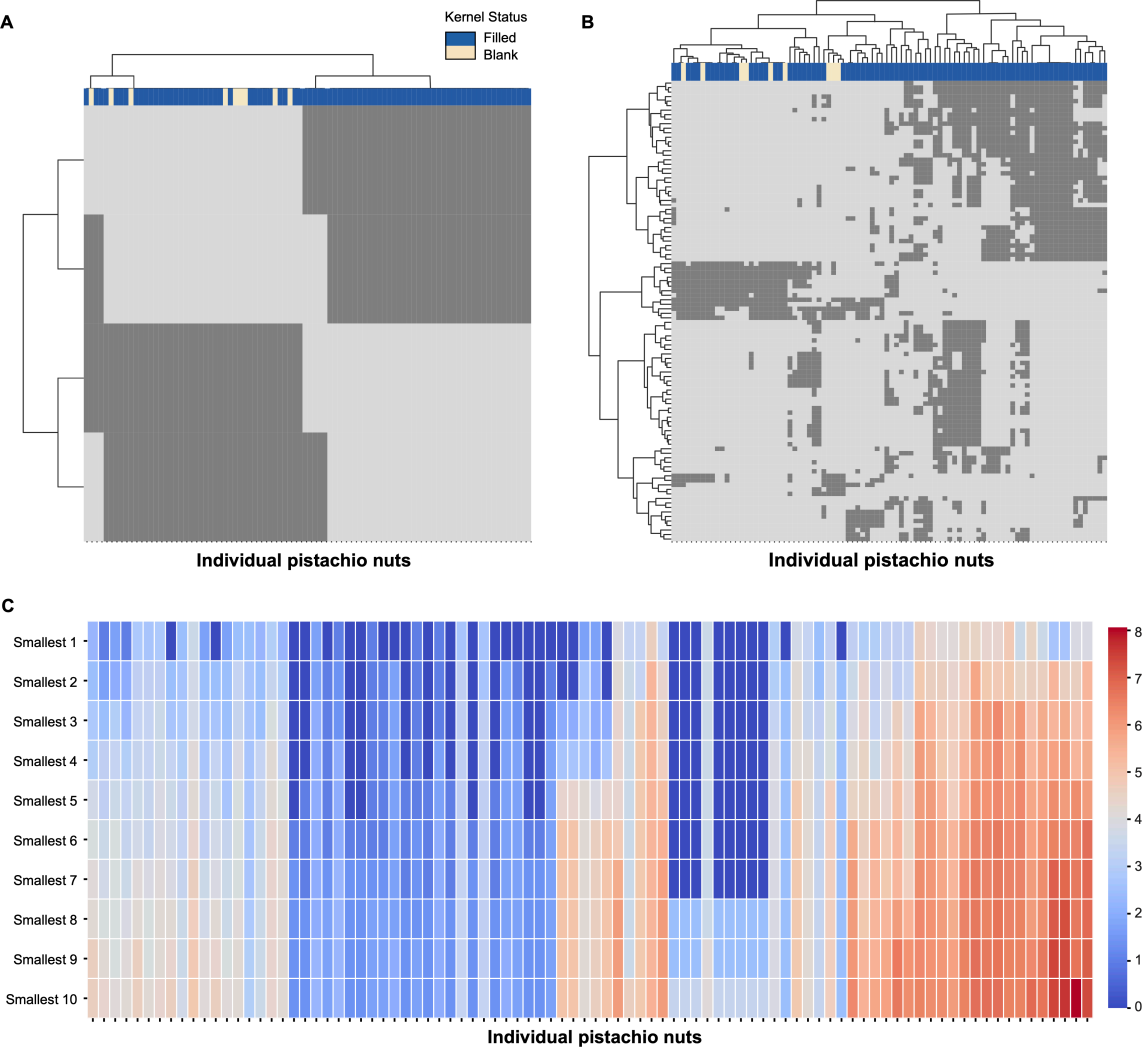
Heatmaps of (A) major 1-feature-categories and (B) major 2-feature-categories at CT1. The specific features on the *y*-axis can be found in electronic supplementary material, table S1. (C) Heatmap of minimum distance among pistachios via heatmap major 2-feature-categories at CT1.

**Figure 15.**
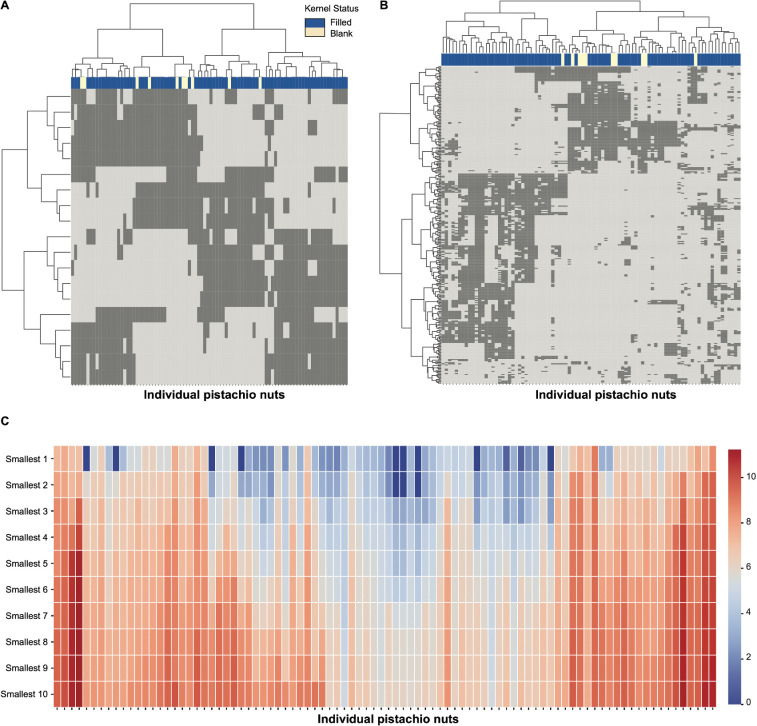
Heatmaps of (A) major 1-feature-categories and (B) major 2-feature-categories at CT2. The specific features on the *y*-axis can be found in electronic supplementary material, table S1. (C) Heatmap of minimum distance among pistachios via heatmap major 2-feature-categories at CT2.

**Figure 16.**
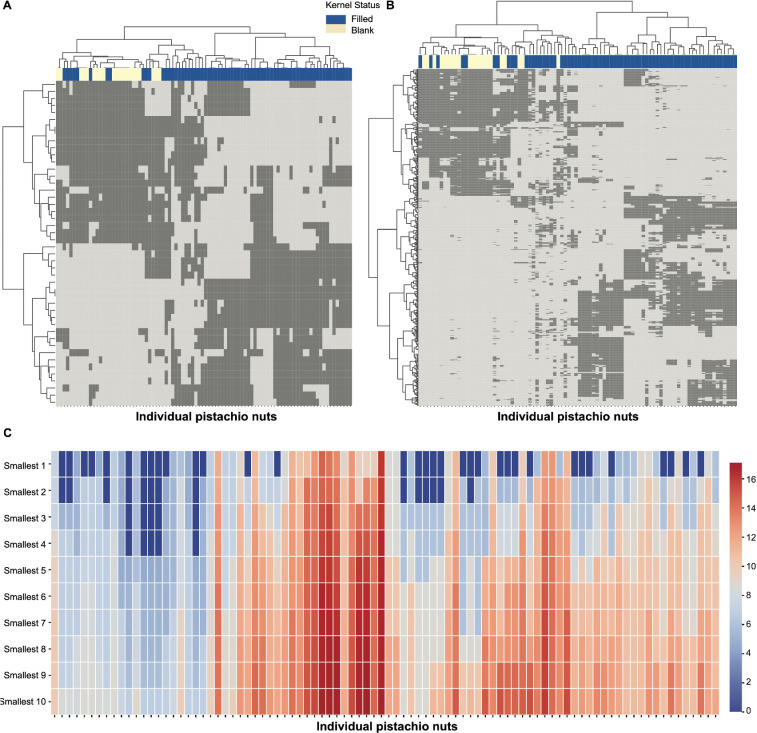
Heatmaps of (A) major 1-feature-categories and (B) major 2-feature-categories at CT3. The specific features on the *y*-axis can be found in electronic supplementary material, table S1. (C) Heatmap of minimum distance among pistachios via heatmap major 2-feature-categories at CT3.

We present CEDA results for each of the three critical time points. At each time point, we identify the significant single‐feature and two‐feature categories and display them as a binary bipartite network in matrix form. In this matrix, the 90 pistachios run along the columns, and the confirmed feature categories (of the chosen order) run along the rows. Each cell is encoded as 1 if that pistachio exhibits the given feature category, or 0 if it does not. Such rearrangement operations were carried out by applying the HC algorithm separately upon all binary row vectors and all binary column vectors, on which the Euclidean distance was defined, and the Ward-D2 module to facilitate distance evaluations between two sets of binary vectors. The resultant block-sustained matrix lattice was called a heatmap. The critical time point-specific heatmap-pair is presented accordingly in figures 14–16.

For each pistachio-specific column, we can read out its presence or absence within which confirmed feature-categories. This binary column-vector is the colour landscape for individual pistachios. Further, the HC-tree superimposed on the column-axis reveals that pistachios shared the same HC-tree branch because of their similarity in individual colour landscapes. This tree geometry gives rise to the neighbourhood system of a heatmap-specific topological pistachio space. Furthermore, as each pistachio is annotated with its kernel’s statuses, filled or blank, we can see the associative patterns from the topological system to colour-marked blank and filled statuses in a collective fashion of colour-bar placed underneath the HC-tree, as shown in figures 14–16.

On the other hand, branches or clusters of the HC-tree seen on the row-axis coupled with branches or clusters of the HC-tree on the column-axis, together, map out the block-system heatmap shown in figures 14–16. Such a block-system sustained heatmap can reveal characteristics of any single cluster or branch of pistachios through its corresponding vertical series of blocks. These traits are closely linked with colour-and-kernel relational knowledge or even intelligence. The essential merits of such computable knowledge and intelligence are tied to addressing pistachio growers’ most important concerns: (i) identifying the nut developmental stage via pistachio images leading to the harvest time; (ii) evaluating the incidence of blanks. We specifically addressed the first concern by identifying which one of the six stages is marked with respect to the three critical time points in the following three subsections. The second concern can be addressed and resolved in tandem with the first.

### 3.1. Heatmap-based topological space and geometric information from CT1 to CT3

We generated three pairs of heatmaps of computed and confirmed major 1-feature-categories and 2-feature-categories with respect to the three critical time points: CT1, CT2 and CT3 in figures 14–16, respectively. Though the information displayed via a heatmap in the three figures explicitly reveals a topological neighbourhood system among all 90 pistachios, it would be even more revealing if we had measurements of the distances of each pistachio to its *K*-nearest neighbours.

We chose K=10 with the idea that these 10 nearest neighbours should fully convey the local geometric information pertaining to each pistachio. This choice can be adjusted when sample sizes are larger than 90. In this study, we report the 10 nearest neighbours based on local geometric information of each of the 90 pistachios based on the major 2-feature-categories across the three critical time points, respectively. These three 10-nearest neighbours based on local geometric information are shown in figures 14C, 15C and 16C. Such 10-nearest neighbours based on local geometric information would play an essential role in identifying a pistachio’s developmental stage only based on its individual image.

The evolving colour changes at three critical time points are particularly noticeable between CT2 and CT3. More detailed colour-changing patterns, which go beyond human visual capabilities, are outlined in table 1 in §2.6 and §3. The visible colour changes from CT1 to CT2 are minimal, mostly observed around the tip area (see figure 2B). By contrast, the colour changes from CT2 to CT3 are pronounced, with most nuts displaying widespread redness. However, some nuts show similar colour properties between CT2 and CT3. The computational results in this section highlight the heterogeneity among nuts.

#### 3.1.1. CT1 results

From the image of a plate of 30 pistachios shown in figure 1C, we can hardly visualize any evident colour distinctions among individual pistachios. So, it is hard to conceive that some major colour proportions extracted from some parts of pistachio hulls are indeed associative with the nut’s blank-vs-filled statuses. However, the human visual capability is limited with respect to the fact that four major 1-feature-categories are indeed discovered; see figure 14A. These features, tip-closest-50-8 and tip-closest-70-8, are of the same major colour code extracted from two portions near the nut tips. What is even more interesting is that the heatmap of a collection of major 2-feature-categories as shown in figure 14B: The right big branch of the HC-tree at 2-branch level is exclusively filled, while the left branch of the HC-tree contains a mixture of filled and blanks. This separation strongly indicates that some colour clues of the blank are somehow captured computationally.

From figure 14B, the largest left (L1) and right (R1) branches are split at the highest internal node of the HC-tree on the column axis. The left (L1) branch contains all the blank pistachios and some filled ones. The the right (R1) branch contains filled pistachios exclusively. These two branches are very distinctively characterized by empty-, sparse- and solid-blocks due to the presence and absence of memberships pertaining to the collection of major 2-feature-categories arranged along the row axis. The left (L1) branch consists of one large empty block and one large sparse block with a near solid block in between them. Pistachios belonging to this branch will certainly have a small distance from the 10 nearest neighbours. This pattern is seen in figure 14C.

The right (R1) branch is further split into two sub-branches: left (R1L2) and right (R1R2), at the second internal node of the HC-tree. The pistachios belonging to the left (R1L2) sub-branch are characterized by one large solid block and one large sparse block. Thus, these pistachios will also have small distances from their 10 nearest neighbours, as seen in figure 14C. In sharp contrast, the pistachios belonging to the R1R2 sub-branch all have relatively large distances towards their 10 nearest neighbours, also seen in figure 14C.

In summary, figure 14C is a graphical display of local geometric information on all pistachios within the topological space. This geometric information is manifested through the heatmap of individual colour landscapes in figure 14B. Such local geometric information will be useful to check whether an unknown pistachio’s developmental stage is within critical time point CT1. This verification can be performed by first finding out its 10 nearest candidate neighbours (NcN) among the 90 pistachios of CT1 as seen in the topological space manifested in figure 14B. Secondly, we judged and decided which of these 10 NcNs would take the unknown one as their 10 nearest neighbours. We built an algorithm to explicitly illustrate and carry out this checking task in the next subsection.

#### 3.1.2. CT2 results

From the image of a plate of 30 pistachios shown in figure 1C, we can see colour seemingly changed from CT1 to CT2 in a uniform fashion. It is still difficult to see colour distinctions among individual pistachios. Nonetheless, there were 21 major 1-feature-categories found, as shown in figure 15A. This heatmap reveals the 10 blank pistachios were spread among the four branches on the HC-tree’s five-branch level. By contrast, these 10 blank nuts are more aggregating together to a great degree in the heatmap of major 2-feature-categories as shown in figure 15B. From this perspective, the heatmap in figure 15B has collectively revealed more information regarding associative patterns from major 2-feature categories towards the kernel’s filled and blank statuses.

Like in CT1, the local geometric information of all 90 pistachios in CT2 is collectively revealed through the distances towards their individual 10 nearest neighbours, as shown in figure 15C.

#### 3.1.3. CT3 results

The image of a plate containing 30 pistachios, shown in figure 1C, reveals significant colour changes from CT2 to CT3, which occur in a notably heterogeneous fashion. Based on their observations, plant scientists and pistachio experts would anticipate that the distinction between blank and filled kernels will become more apparent. This expectation aligns with the fact that the 21 blank pistachios are clustered together, as illustrated in the heatmaps of the major 1-feature categories in figure 16A. This clustering pattern becomes even more pronounced in the heatmap of the major 2-feature categories presented in figure 16B. Such a clear aggregation pattern will allow for more accurate evaluations of the prevalence of blank pistachios in CT3 compared with CT2, where the clustering is less evident.

Similarly, based on the heatmap of major 2-feature-categories in figure 16B, the local geometric information via distances of 10 nearest neighbours of the 90 pistachios in CT3 is shown in figure 16C. It is reiterated that a branch or cluster of pistachios, characterized by a vertical series of clearly either empty or solid blocks, would have rather small distances towards their nearest neighbours.

### 3.2. Algorithm for determining pistachio developmental stage

The series of three heatmaps corresponding to the critical time point axes provides significant advantages for both plant scientists and pistachio growers. When analysing a batch of pistachios, these heatmaps can help make informed decisions about harvest timing and assess the prevalence of blanks using two established protocols, which will be detailed in the next subsection.

This section illustrates the construction of an algorithm to determine the nut developmental stage based on a heatmap of the two major feature-categories, as shown in figure 16. The algorithm provides each of the 90 pistachios with a binary column vector. This vector indicates the presence or absence of membership across all confirmed major two feature-categories for each pistachio. This binary presence–absence vector effectively describes the colour characteristics of each pistachio through two interacting features. Therefore, it is referred to as the *individual colour landscape of the nut*. Collectively, these individual colour landscapes enable us to rearrange and visualize the similarities among the 90 pistachios. This computable similarity also facilitates the application of the mathematical concept of topology.

In mathematics, a topology is defined by a neighbourhood system. Here, the neighbourhood system is simply defined via the Euclidean distance upon the 90 binary column vectors. Hence, each heatmap would be viewed as a graphic display of topology defined on the 90 pistachios’ colour landscapes. This neighbourhood system plays an essential role in determining the groupings of pistachios. That is, a neighbourhood of a column vector is used to decide whether an unknown pistachio is similar enough by being inside its neighbourhood.

As such, a map of similarity is more or less revealed through the block patterns embraced by each heatmap. In particular, the algorithm developed here is based on the three heatmaps of major 2-feature-categories as shown in figures 14B, 15B and 16B. The intuition behind this algorithm is summarized as follows:

‘If an unknown subject is to be found within the neighbourhood of a known subject, then its local geometric information, which is revealed by its 10 ranked distances to its 10 nearest candidate neighbours (NCN), should be very close to the local geometric information of this known subject reflected by its 10 ranked distances to its own nearest neighbours, such as shown in figures 14C, 15C and 16C)’.

This is one simple way of checking by visualizing whether an unknown subject is within a known subject’s neighbourhood. That is, they have to share the same local geometric information. From this perspective, a neighbourhood system typically embraces size heterogeneity. This is evident from the HC-tree superimposed on the column axis. By having a horizontal cut at a given tree height, a collection of branches: large or small in size, is generated.

#### 3.2.1. Growth stage determining algorithm

— A ’Golden Hills’ pistachio, say x, of an unknown developmental stage, would also have three individual colour landscapes pertaining to the three critical time point specific three heatmaps of major 2-feature-categories as shown in figures 14B, 15B and 16B.
— At ct-th critical time point, a specific heatmap with ct=1,2,3*,* a group of K nearest candidate-neighbours (NcN) are identified: $CandiG_{ct}=\left\{z_{1}^{\left(ct\right)},z_{2}^{\left(ct\right)},..z_{K}^{\left(ct\right)}\right\}$.
— Each candidate-neighbour $z_{k}^{\left(ct\right)}$ with k=1,..,K has its neighbourhood denoted as $N[z_{k}^{\left(ct\right)}]$ with its size denoted as $|N[z_{k}^{\left(ct\right)}]|$. We calculate the maximum among the sizes of the intersection $N[z_{k}^{\left(ct\right)}]⋂CandiG_{ct}$ relative to $|N[z_{k}^{\left(ct\right)}]|$ as
$$Odds_{G_{ct}}=argmax_{k=1,.,K}\frac{|N[z_{k}^{\left(ct\right)}]⋂CandiG_{ct}|}{|N[z_{k}^{\left(ct\right)}]|}.$$
— With the triplet vector $\left(Odds_{G_{1}},Odds_{G_{2}},Odds_{G_{3}}\right)$, we will be able to decide regarding to x’s growth stage: (i) before critical time point 1; (ii) at critical time point 1; (iii) after critical time point 1, but before critical time point 2; (iv) at critical time point 2; (v) after critical time point 2 but before critical time point 3; (vi) at critical time point 3.

We first illustrate the use of the first part of the growth stage determining (GSD)-Algorithm on a pistachio x being CT2-A1-full-22 from CT2 and its K nearest candidate neighbours on the colour landscape of CT3. Specifically, we choose K=10, and these 10 nearest-candidate-neighbours (NcN) (K=10): $CandiG_{3}$, selected among the 90 pistachios, are ranked and arranged along the column-axis in table 6. The 10 ranked corresponding distances are given in the 11th row. Upon the kth column, we list the 10 ranked distances of nearest neighbours of $z_{k}^{\left(3\right)}$ with k=1,2,...,10. By comparing these 10 distances with the distance at the bottom row, we count whether this x receives a vote or not. We found that, across all columns of table 6, we see that x (CT2-A1-full-22) receives zero votes. That is, the x’s $Odds_{G_{3}}=0$. In other words, this pistachio CT2-A1-full-22 from the CT2 is an outlier based on the colour landscape of CT3.

**Table 6.** An illustrative example of GSD-Algorithm: 10 nearest candidate neighbours (NcN) of a pistachio (CT2-A1-full-22) from CT2 within the topological colour landscape of CT3 and these 10 NcNs' 10 nearest neighbours.

NN-vs-NcN	A5-full-22	A7-full-9	A7-full-25	A13-full-16	A13-full-23	A5-full-1	A5-full-2	A13-full-25	A5-full-10	A5-full-28
1	0.00	0.00	0.00	0.00	0.00	4.24	0.00	0.00	5.10	5.39
2	0.00	0.00	0.00	0.00	0.00	4.24	4.24	4.24	5.10	5.39
3	0.00	0.00	0.00	0.00	0.00	4.24	4.24	4.24	5.10	5.39
4	0.00	0.00	0.00	0.00	0.00	4.24	4.24	4.24	5.10	5.39
5	4.24	4.24	4.24	4.24	4.24	4.24	4.58	4.58	5.10	5.39
6	4.58	4.58	4.58	4.58	4.58	4.58	4.58	4.58	5.291	6.71
7	4.58	4.58	4.58	4.58	4.58	6.24	4.58	4.58	6.48	6.93
8	5.10	5.10	5.10	5.10	5.10	6.24	6.24	6.24	6.71	6.93
9	5.39	5.39	5.39	5.39	5.39	6.48	6.71	6.71	6.71	7.28
10	6.24	6.24	6.24	6.24	6.24	6.71	6.71	6.71	7.28	8.00
CT2-A1-full-22	9.27	9.27	9.27	9.27	9.27	9.90	10.15	10.15	10.39	10.44

**Figure 17.**
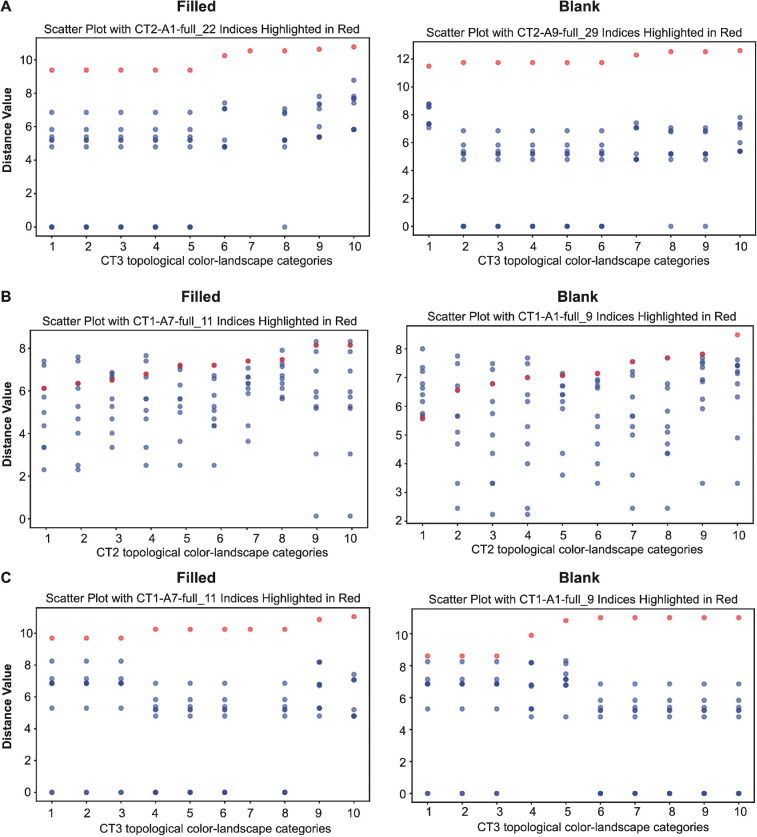
(A) A graphic display of table 6. A filled pistachio nut CT2-A1-full22's and a blank pistachio nut CT2-A9-full29's geometric information within the topological colour landscape of CT3. (B) Two graphic displays of geometric information of a filled pistachio CT1-A7-full11 and a blank pistachio CT1-A1-full9 within the topological colour landscape of CT2. (C) Two graphic displays of geometric information of a filled pistachio CT1-A7-full11 and a blank pistachio CT1-A1-full9 within the topological colour landscape of CT3.

The graphic display of table 6 is shown in figure 17A. By contrast to the filled pistachio CT2-A1-full-22, a blank pistachio CT2-A9-full-29’s relational information within the topological colour landscape of CT3 is graphically displayed on the right. Likewise, this panel reveals that this blank pistachio is also far from the topological space of CT3.

Next, we show two graphic displays of geometric information of two pistachios: one filled and one blank, from the CT1 within the topological colour landscape of CT2 and CT3 in figure 17B,C, respectively. Again, the two bivariate $\left(Odds_{G_{2}},Odds_{G_{3}}\right)$ are calculated as (0,0). This demonstration is essential because the error of mistaking a pistachio from CT1 as being from CT2 or CT3 could incur tremendous costs to growers.

### 3.3. Inferential protocols for identifying developmental stages leading to harvest and evaluating blank incidence

The original motivation for the colour study of pistachio nuts comes from the quest to pinpoint the growth stage of a pistachio through its image and inferring its likelihood of carrying a filled or blank nut. The solutions to these two quests would lead to a protocol for determining harvest time based on GDD calculation and for evaluating the blank prevalence rate. In this subsection, we applied the GSD-Algorithm to construct these two protocols.

#### 3.3.1. A protocol for determining harvest time

When GSD-Algorithm is applied to a batch of pistachios, we summarize results based on the collection of triplet vectors $\left(Odds_{G_{1}},Odds_{G_{2}},Odds_{G_{3}}\right)$ according to the following protocol. Since each unknown pistachio in this batch is computed with specific triplet vectors $\left(Odds_{G_{1}},Odds_{G_{2}},Odds_{G_{3}}\right)$ that summarize its relative geometric relations based on the three heatmaps of 2-feature-categories together with its corresponding 10 NCNs’ local geometric information with respect to CT1, CT2 and CT3, respectively, then, the ordering pattern of these three odds across all unknown pistachios in the batch will shed light on the potential developmental stage.

— Before-CT1:
  majority of unknown pistachios received their triplets $(Odds_{G_{1}},Odds_{G_{2}},Odds_{G_{3}})=\left(0,0,0\right)$;
— At-CT1:
  majority of unknown pistachios received their triplets $(Odds_{G_{1}},Odds_{G_{2}},Odds_{G_{3}})=\left(1,0,0\right)$;
— Between CT1 and CT2:
  majority of unknown pistachios received their triplets $(Odds_{G_{1}},Odds_{G_{2}},Odds_{G_{3}})=\left(\delta ,1-\delta ,0\right)$;
— At-CT2:
  majority of unknown pistachios received their triplets $(Odds_{G_{1}},Odds_{G_{2}},Odds_{G_{3}})=\left(0,1,0\right)$;
— Between CT2 and CT3:
  majority of unknown pistachios received their triplets $(Odds_{G_{1}},Odds_{G_{2}},Odds_{G_{3}})=\left(0,\delta ,1-\delta \right)$;
— At-CT3:
  majority of unknown pistachios received their triplets $(Odds_{G_{1}},Odds_{G_{2}},Odds_{G_{3}})=\left(0,0,1\right)$.

It is worth reiterating that from the figure 17, we can infer that this protocol passes the reliability check. From a computational perspective, this collection of $\left(Odds_{G_{1}},Odds_{G_{2}},Odds_{G_{3}}\right)$ provides the foundation for any idiosyncratic protocols to be developed and adopted by individual growers. Further, it is necessary to ensure a grower’s orchard-specific variation is properly accommodated.

#### 3.3.2. A protocol for evaluating the blank prevalence rate

Besides the growth stage before CT1, we can evaluate the incidence of blank nuts via the following protocol based on the collection of results after applying the above protocol for harvest time upon a batch of pistachios.

— At each one of the five stages in the protocol for harvest time, except before-CT1, an unknown pistachio is associated with a known pistachio, which is seen being located at a neighbourhood (branch) with a visible rate of blank. We annotate this unknown pistachio with its identified known neighbour’s rate of blank.
— We built a histogram based on the collection of annotated rates of blank to represent the distribution of the rate of blank among this batch of unknown pistachios.

This resultant distribution would be very informative for growers regarding the blank rate for their orchards in the growing season. Since the rates of blank have distinct precisions across different HC-tree branches, as seen in figures 14B, 15B and 16B, we calculate for an estimation of the size of a batch of unknown pistachios. It is necessary to first determine the proportions of unknown pistachios falling into all branches. That is, we need to make sure that all branch-specific estimated rates are simultaneously achieving the required precision.

## 4. Discussion

This study demonstrates how a physiologically informed, image-based computational approach can be used to extract interpretable and biologically meaningful information from pistachio hull colour data. By designing a feature construction protocol rooted in domain knowledge, we translated spatial colour patterns into structured, multivariate data representations. The subsequent application of CEDA enabled the extraction of reliable, high-order associative relationships between colour features and kernel fill status across developmental stages.

### 4.1. Computational foundations of the image-based protocol

Our image-based data analysis protocol involved two key computational developments. The first begins with processing each nut’s colour image: identifying its tip, extracting 56 major colour proportions across seven distinct sections of the hull, and constructing structured databases at each of the three critical time points. Every step in this process relied on domain expertise. Image processing choices, including selecting representative nuts for building the eight-colour spectrum (figure 3), were informed by expert knowledge to ensure inclusivity of both filled and blank nuts across developmental stages.

Feature construction, transforming an image into a structured vector of measurements, was a critical step that encoded biological insight. The seven spatial sections were selected based on prior understanding of tip-to-base colour patterns associated with kernel filling. Scientific analysis benefits when data representations align with known biological structures, as this coherence enables meaningful discovery beyond existing knowledge. Interpretability of features was a guiding principle. While automated image-based feature selection techniques exist, they often lack transparency, limiting their utility in scientific applications. To underscore this point, we compared our expert-guided approach with three standard machine learning methods. Our response variable was the binary kernel status (filled or blank), another choice rooted in biological relevance. With 56 covariate features and this designated response, we established a structured response–covariate framework to model the underlying biological system.

The second computational development involved adopting CEDA as our primary analytical paradigm [11,12]. For categorical outcomes like kernel status, contingency tables provide a natural and interpretable platform to examine associations. Standard parametric approaches (e.g. correlation via linear regression) are often inappropriate in this context. CEDA excels by enabling exploration of higher-order interactions, such as simultaneous colour patterns across multiple hull regions. Importantly, each association must pass a reliability check, ensuring it is statistically valid given the finite sample size. This check is based on two types of simulated randomness—observable and null—following the framework advocated by Kolmogorov [10]. From this, we constructed ensembles of contingency tables: one mimicking observed data, the other reflecting null distributions. Each row-wise association was evaluated using overlapping odds distributions to minimize both Type-I and Type-II errors.

Selected associations were encoded as binary vectors denoting nut membership, allowing the construction of a bipartite matrix: nuts (columns) versus selected feature associations (rows). The binary nature of this matrix permitted Euclidean distance-based clustering. Each column, termed a nut’s individual colour-character landscape, contributed to a topological space of colour profiles. Hierarchical clustering applied to both axes yielded a block-structured heatmap—a multiscale visualization of associations. Within this heatmap, clusters of nuts could be characterized by patterns of colour associations. When annotated with kernel status, these clusters provided interpretable insights into classification, allowing evaluation of accuracy at both global and local scales—an approach distinct from traditional classification models. We generated heatmaps from first- and second-order feature associations at each of the three critical time points (90 nuts per time point). These visualizations not only revealed developmental shifts in hull colouration but also enabled classification across seven temporal regions: the three critical time points and four intervals between them.

### 4.2. Environmental and practical considerations

From a physiological perspective, hull colouration is influenced not only by maturation but also by environmental conditions. For example, increased sunlight exposure is known to enhance red pigmentation in the hull [13]. While this study accounted for microclimate variation through diverse sampling across canopy positions, further improvements in standardization are possible. Expanding the dataset across multiple years and orchard locations would strengthen the generalizability and robustness of the protocol.

Taken together, these findings underscore the value of a data-driven yet interpretable analytical framework. This integrative approach offers more than a classification tool; it demonstrates how structured colour data can be used to bridge physiological traits with actionable outcomes in harvest timing and quality control.

### 4.3. Application in commercial orchard settings

The most direct application of our image-based analysis protocol in commercial settings is as a tool to accelerate, standardize, and enhance current harvest-timing assessments. Growers typically evaluate ripening by visually assessing hull colour and hull breakdown, an indicator of readiness defined by the ease with which the hull detaches from the shell under pressure along the longitudinal axis [2]. Our approach replaces this subjective method with a more objective and scalable alternative: growers can photograph nuts under standardized lighting conditions and upload the images to an online interface. This interface, powered by our GSD-Algorithm, can accurately estimate the optimal harvest time and predict the incidence of blank nuts.

Beyond standardization and time savings, a key advantage of adopting this method will be its ability to provide harvest predictions a few weeks earlier than the conventional sensory-based approach. By detecting physiological changes through colour patterns, the algorithm can support earlier decision-making, thereby reducing nut defects and improving harvest logistics and economic outcomes.

To support field implementation, we also developed a practical image collection protocol tailored for growers (see electronic supplementary material, protocol S1). We recommend using a smartphone or GoPro camera mounted on an extension pole with a remote shutter and a colour reference ring for white balancing. Pistachio clusters are photographed directly on the tree from two slightly different angles to improve the reliability of colour analysis. We suggest growers select 10−20 clusters from representative orchard locations to capture local variability. After images are submitted, analysts can extract hull colour features from multiple nuts per cluster and classify them based on their developmental stages relative to the three critical time points. The results are returned as a summary report that helps growers understand the current ripening status of their orchard.

With further development, the method could be adapted for in-field application through a smartphone app capable of analysing nuts on the tree directly. This would simplify the process for growers by reducing the number of required steps. A primary challenge in this context will be accounting for variations in natural lighting. However, advances in image normalization and calibration could address these constraints.

Finally, our protocol could be integrated into existing fixed or mobile remote sensing frameworks used to monitor fruit yield, phenology and quality. Such integration would allow for large-scale monitoring of nut ripening and blank nut incidence across entire orchards, with significant potential to improve management decisions and resource allocation.

## 5. Conclusions

This study presents two practical protocols for plant scientists and nut growers: one for determining the optimal harvest time for pistachios and another for assessing the incidence of blank nuts. The protocols are designed to provide growers with essential decision-making information, enabling them to make informed choices without needing to explore the technical details. As data analysts, we acknowledge our responsibility to ensure that decisions regarding critical aspects of pistachio development are sound, as growers ultimately face the consequences of these decisions. There is also considerable potential for future work to standardize the model and expand its applicability. Specifically, incorporating data from additional years and a wider range of orchard locations would help capture greater environmental and management variability. Additionally, establishing a well-defined sampling protocol, including clear guidelines for sample size and location, would greatly benefit both growers and researchers.

The GSD-Algorithm uses a series of heatmaps to determine a pistachio’s maturity stage by identifying its status at one of three critical time points or between them. This capability is essential to the algorithm’s topological structure and would help growers determine optimal harvest timing. Another significant advantage of the GSD-Algorithm is its ability to estimate the prevalence of blank nuts. Each of the 90 pistachios is labelled with a binary ID: filled or blank. By analysing the binary IDs of neighbouring pistachios, the algorithm can evaluate the likelihood of unknown pistachios’ IDs based solely on their images. This application is especially useful as growers could photograph marked branches in their orchards without needing to cut them.

Moreover, the heatmaps provide critical insights into the overall prevalence of blank nuts in the orchard. By selecting clusters from the hierarchical clustering tree along its column axis, we calculate cluster-specific blank prevalence rates and derive a weighted overall estimate. This predictive approach differs from simply predicting the status of individual pistachios.

While the protocol was tailored for pistachios, it could be adapted for use with other cultivars and crops where changes in hull or fruit colour indicate maturity or ripening. For example, this approach could be adapted to almonds, peaches or mangoes, taking into account the unique characteristics of each crop. This versatility highlights the broader potential of the model for advancing harvest management and quality evaluation across different agricultural systems.

## Data Availability

The data and Python scripts that support the findings of this study are available on Zenodo [14] and in the electronic supplementary material of this article. Additional data are available from the corresponding author upon reasonable request. Electronic supplementary material is available online [15].
